# Hormone therapy use and the risk of acute kidney injury in patients with prostate cancer: a population-based cohort study

**DOI:** 10.1038/s41391-021-00348-x

**Published:** 2021-03-26

**Authors:** Chris R. Cardwell, Joe M. O’Sullivan, Suneil Jain, Blánaid M. Hicks, Paul A. Devine, Úna C. McMenamin

**Affiliations:** 1 Centre for Public Health, Queen’s University Belfast, Belfast, Northern Ireland UK; 2 Centre for Cancer Research and Cell Biology, Queen’s University Belfast, Belfast, Northern Ireland UK; 3 Radiotherapy Department, Cancer Centre, Belfast City Hospital, Belfast, Northern Ireland UK; 4 Regional Nephrology and Transplant Unit, Belfast City Hospital, Belfast, UK

**Keywords:** Cancer epidemiology, Cancer therapy, Outcomes research

## Abstract

**Background:**

Hormone therapy is widely used in prostate cancer. However, studies have raised concerns that hormone therapy, particularly the use of gonadotropin-releasing hormone agonists, could increase the risk of acute kidney injury.

**Methods:**

Men newly diagnosed with non-metastatic prostate cancer, from 2012 to 2017, were identified from the Scottish Cancer Registry. A matched comparison cohort of prostate cancer-free men was also identified. Hormone therapy use was determined from the Prescribing Information System in Scotland. The primary outcome was hospitalisations with acute kidney injury taken from Scottish hospital records (SMR01) up to June 2019. Time-dependent Cox regression models were used to calculate hazard ratios (HRs) and 95% confidence intervals (CIs) for acute kidney injury by hormone therapy use.

**Results:**

The prostate cancer cohort contained 10,751 patients followed for 41,997 person years, during which there were 618 hospitalisations with acute kidney injury. Prostate cancer patients had higher rates of acute kidney injury compared with cancer-free controls (adjusted HR = 1.47 95% CI 1.29, 1.69). However, prostate cancer patients currently using hormone therapy (adjusted HR = 1.14 95% CI 0.92, 1.41), including gonadotropin-releasing hormone (GnRH) agonists (adjusted HR = 1.13 95% CI 0.90, 1.40), did not appear to have a marked increase in acute kidney injury compared with prostate cancer patients not using hormone therapy after adjusting for potential confounders.

**Conclusions:**

In our cohort, there was little evidence that gonadotropin-releasing hormone agonists were associated with marked increases in acute kidney injury.

## Introduction

Androgen deprivation therapy was initially used for advanced prostate cancer [1] but is increasingly being used in more localised disease [2, 3]. However, the marked reductions in testosterone, caused by androgen deprivation therapy, are associated with various side effects including fractures, diabetes and cardiovascular disease [4].

Recently concerns have been raised that androgen deprivation therapy could also increase the risk of acute kidney injury. Various potential mechanisms have been proposed [5, 6]. For instance, androgen deprivation therapy increases dyslipidemia and hyperglycemia which may disrupt glomerular function [7]. Androgen deprivation therapy reduces testosterone and testosterone may directly protect vasodilation of renal vessels [8]. In addition, androgen deprivation therapy may increase the risk of various cardiovascular disease, which along with treatments for cardiovascular disease, could increase acute kidney injury risk [9, 10]. To date, only two studies [5, 6] have investigated hormone therapy use and acute kidney injury in humans. A UK study [5] observed a large (150% increase) in acute kidney injury with current hormone therapy use whilst a US study [6] observed a comparatively small 24% increase with any use of gonadotropin-releasing hormone (GnRH) agonists. Further research is warranted not only because the magnitude of associations was so markedly different in these studies but also because these studies had weaknesses (for instance, one [5] could not adjust for cancer stage whilst the other [6] could not determine dose or duration of androgen deprivation therapy use).

Therefore, we investigated the association between hormone therapy use and acute kidney injury in a contemporary population-based prostate cancer cohort from Scotland.

## Subjects and methods

### Data sources

The following linked databases were utilised [11]: the Scottish Cancer Registry; the Scottish National Prescribing Information System (PIS), which captures all community dispensed medications in Scotland; the General/Acute Inpatient and Day Case dataset (SMR01), which captures hospital diagnoses and operations and has high accuracy for various diagnoses [12]; the Outpatient Attendance dataset (SMR00), which captures diagnoses and procedures from outpatient clinics; and, the National Records of Scotland Death Records which captures date and cause of death. These databases covered Scotland from January 1999 to June 2019, apart from the PIS which was available from January 2009 to June 2019. Linkages between data sources were conducted using the Community Health Index number [11]. The study was approved by the Privacy Advisory Committee of the National Health Service National Services Scotland (number: 1617-0374).

### Study design

A cohort of men newly diagnosed with non-metastatic (M stage 0) prostate cancer (ICD10, International Classification of Diseases 10th revision, code C61) between January 2012 and December 2017, was identified from the Scottish Cancer Registry. Patients were excluded if they: had a previous cancer diagnosis (apart from non-melanoma skin cancer or in situ tumours); had inconsistent dates (specifically a record of hormone therapy or radical prostatectomy more than 6 months before cancer diagnosis); or, had a renal disease diagnosis (including acute kidney injury, defined later, or chronic kidney disease, based upon ICD10 codes [13]) prior to prostate cancer diagnosis.

A separate cohort of cancer-free controls was also selected. One population-based control was randomly selected, without replacement, using the Community Health Index database, matched on year of birth to each patient with prostate cancer. The index date in the controls was defined as the date of prostate cancer diagnosis in their matched case.

The primary outcome was hospitalisation for acute kidney injury identified by the ICD10 code of N17 as the main or secondary condition, as previously defined [5, 14], in SMR01. Thus, prostate cancer patients were followed from the date of prostate cancer diagnosis (and controls were followed from the index date) to the earliest of the date of first acute kidney injury, date of death, date of leaving Scotland or June 2019.

### Exposure

Hormone therapy consisted of GnRH agonists (including goserelin, leuprorelin, triptorelin and histrelin), the GnRH antagonist degarelix, oral anti-androgens (including bicalutamide, enzalutamide, flutamide and cyproterone acetate), estrogens and orchidectomy. Medical hormone therapy was identified from dispensed medications from the PIS. The pack size and strength were used to calculate days of use based upon the daily defined doses (DDDs) from the World Health Organisation[15]. The Scottish Cancer Registry provided data on initial curative radiotherapy. Orchidectomy (ICD10 codes N051, N052, N061 and N063 [16]) and radical prostatectomy (ICD10 code M61[16]) were taken from SMR01.

### Confounders

The Scottish Cancer Registry provided Gleason score and stage (based upon pathological stage, where recorded, or clinical stage). Comorbidities from the Charlson comorbidity index (specifically acute myocardial infarction, congestive heart failure, peripheral vascular disease, cerebrovascular accident, pulmonary disease, connective tissue disorder, peptic ulcer, liver disease, diabetes, diabetes complications, paraplegia, renal disease and severe liver disease, using previous ICD codes [13]) were identified before cancer diagnosis from hospital admissions data (SMR00 and SMR01). The following medications were determined from PIS records in the year prior to diagnosis: non-steroidal anti-inflammatory drugs, aspirin, beta-blockers, angiotensin-converting enzyme inhibitors, angiotensin II receptor blockers (ARBs), diuretics, statins, warfarin, digoxin, clopidogrel, dipyridamole, nitrates, insulin, sulfonylureas, metformin, tamsulosin and other diabetic medications. Deprivation level was determined from the postcode of residence using the 2009 Scottish Index of Multiple Deprivation [17].

### Statistical analysis

An initial analysis was conducted comparing acute kidney injury in prostate cancer patients to all population-based controls using Cox regression models to calculate hazard ratios (HRs) and 95% confidence intervals (CIs) adjusting for age, deprivation, comorbidities (stated above) and other medications (stated above).

**Fig. 1:**
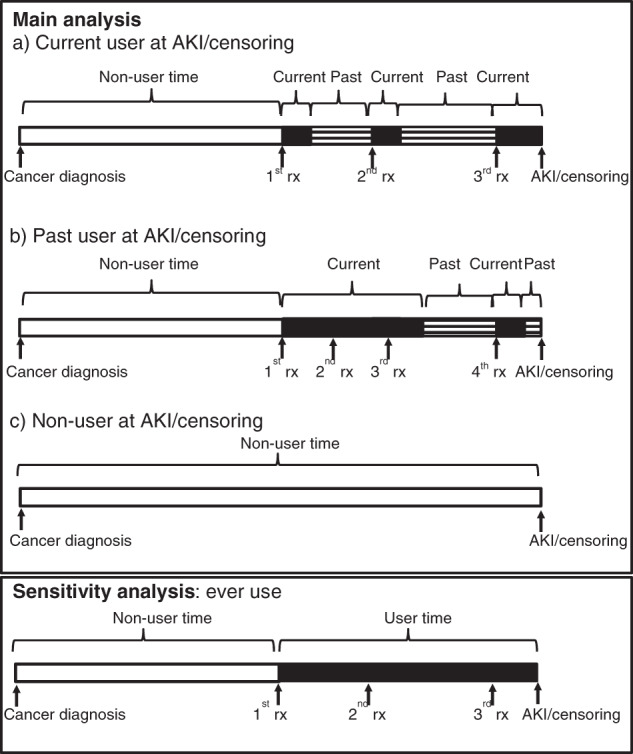
Figure illustrating the study design. Exposure categories are shown for the main analysis in the top panel (including current (**a**), past (**b**) and non-users (**c**)) and for the senstivity analysis in the lower panel.

In prostate cancer patients, current use of hormone therapy was modelled as a time-varying covariate. Patients became a user upon the date of each hormone therapy prescription, and remained a user for the duration of the prescription, based upon the daily defined dose, plus a residual effect. The duration of the residual effect was taken from a previous study [5] and was 3 months for GnRH agonists and antagonists and 1 month for anti-androgens and estrogens. The exposure period from the end of one prescription to the start of the next was allocated to past use. A diagram explaining the study design is shown in Fig. 1. Patients receiving orchidectomy were considered exposed from the date of orchidectomy until the end of follow-up. Cox regression models were then used to calculate HRs and 95% CI for acute kidney injury comparing hormone therapy current use and past use to non-users. The main model contained year of diagnosis, age, deprivation, cancer treatment (radiotherapy and radical prostatectomy as time-varying covariates), comorbidities (stated above, in the year prior to diagnosis) and other medications (stated above, in the year prior to diagnosis). Separate analyses were conducted additionally adjusting for T stage and N stage, including those with stage X [unstaged or stage unknown] as a separate category (corresponding to 3% and 6%, respectively). Analyses were repeated separately for GnRH agonists, the GnRH antagonist degarelix and either GnRH agonists or antagonists.

Additional analyses were conducted. First, analyses were repeated using the outcome of acute kidney injury based solely upon the main condition within hospital records. Second, analysis was repeated using the outcome of hospitalisation or death from acute kidney injury. Third, analyses were conducted introducing a lag of 90 days. Fourth, analyses were repeated assuming a 1-year residual effect for GnRH agonists and analyses were repeating halving all residual effects to 45 days for GnRH agonists and antagonists and 15 days for anti-androgens and estrogens. Fifth, analyses were repeated in patients with M stage 0 or X and separate analysis was repeated including all prostate cancer patients. Sixth, an analysis was conducted restricted to patients with localised prostate cancer (T stage 1 or 2, N stage 0 and M stage 0). Seventh, analyses were repeated censoring individuals on date of radical prostatectomy and/or radiotherapy. Eighth, the analysis was repeated not adjusting for radical prostatectomy and radiotherapy, to avoid the risk of adjusting for an intermediate on the causal pathway[18]. Ninth, an analysis was conducted additionally adjusting for Gleason score, overall and in patients with localised prostate cancer.

Finally, an analysis was conducted based upon any use of hormone therapy with individuals considered users from first use until the end of follow-up. A similar exposure-response analysis was conducted with patients deemed non-users before first use, a short-term user from this time to 365 days of use (based upon DDDs), and a long-term user from then on. All analyses were conducted using STATA 16 (StataCorp, College Station, TX, USA) and STATA code is available (upon request).

## Results

**Fig. 2:**
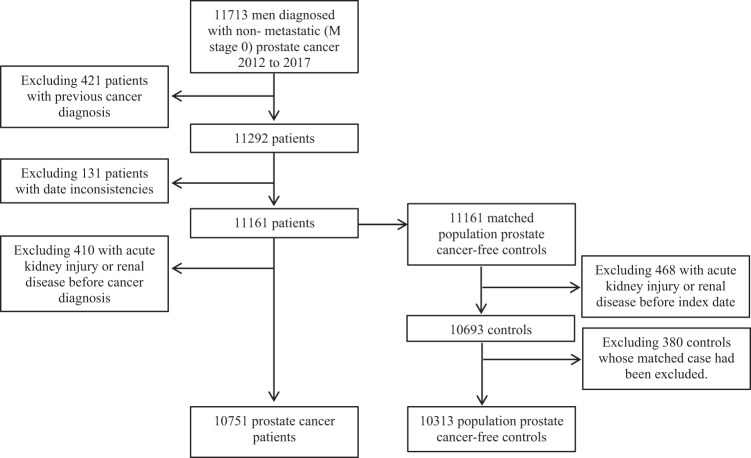
**Flow diagram of selection of prostate cancer patients and matched cancer-free controls.**

Figure 2 shows the patient selection. Overall, 10,751 patients with non-metastatic prostate cancer were followed for 41,997 person years in whom there were 618 hospitalisations with acute kidney injury (15 per 1000 person years).

In total 10,313 population-based controls were identified, including 485 hospitalisations with acute kidney injury, corresponding to a 47% increase in acute kidney injury in non-metastatic prostate cancer patients compared with controls (HR = 1.47, 95% CI: 1.29, 1.69 after adjustment for age, deprivation, medication use and comorbidities).

### Patient characteristics

Hormone therapy users, compared with non-users, were older and had markedly higher T stage, N stage and Gleason score, higher rates of radiotherapy and lower rates of radical prostatectomy (see Table 1). Hormone therapy users also had slightly higher rates of comorbidities and medication use.

**Table 1:** Characteristics of prostate cancer patients by any use of hormone therapy.

	Hormone therapy	
	Non-users	Users^a^
Year of diagnosis		
2012–13	1417 (30%)	1847 (31%)
2014–15	1526 (32%)	2071 (35%)
2016–17	1810 (38%)	2080 (35%)
Age at diagnosis		
<60	986 (21%)	528 (9%)
60–69	2180 (46%)	2134 (36%)
70–79	1259 (26%)	2833 (47%)
90	328 (7%)	503 (8%)
Deprivation		
1st fifth (least deprived)	522 (11%)	829 (14%)
2nd fifth	818 (17%)	1055 (18%)
3rd fifth	969 (20%)	1269 (21%)
4th fifth	1115 (23%)	1425 (24%)
5th fifth (most deprived)	1307 (27%)	1407 (23%)
Radiotherapy (any time)	711 (15%)	3912 (65%)
Radical prostatectomy (any time)	1656 (35%)	297 (5%)
Hormone therapy during follow-up^b^		
GnNRH alone or with anti-androgens		5303 (88%)
Degarelix alone		74 (1%)
Anti-androgens alone		506 (8%)
Orchidectomy alone or with other treatments		12 (0%)
Estrogen alone or with other treatments		9 (0%)
Other combinations		94 (2%)
Gleason score		
≤6	2324 (49%)	746 (12%)
7	1899 (40%)	2757 (46%)
8	170 (4%)	765 (13%)
9	109 (2%)	1259 (21%)
10	5 (0%)	88 (1%)
Missing	246 (5%)	383 (6%)
T stage		
1	851 (18%)	309 (5%)
2	2914 (61%)	2239 (37%)
3	859 (18%)	2991 (50%)
4	20 (0%)	281 (5%)
X	109 (2%)	178 (3%)
N stage		
0	4438 (93%)	4929 (82%)
1	55 (1%)	714 (12%)
X	260 (5%)	355 (6%)
Selected mediations (in year before diagnosis)		
Aspirin	857 (18%)	1484 (25%)
ACE inhibitor	1097 (23%)	1719 (29%)
Statins	1754 (37%)	2738 (46%)
Tamsulosin	1377 (29%)	1810 (30%)
Selected comorbidities (in year before diagnosis)		
Myocardial infarction	146 (3%)	325 (5%)
Heart failure	99 (2%)	191 (3%)
Stroke	132 (3%)	223 (4%)
Diabetes	193 (4%)	336 (6%)

^a^Hormone therapy at any time after diagnosis.

^b^Treatments used at any time during entire follow-up period. Combinations of treatments not necessarily used simultaneously.

### Androgen deprivation therapy and acute kidney injury

Table 2 shows that there was a marked increase in acute kidney injury with the current use of hormone therapy compared with no hormone therapy use (HR = 1.96, 95% CI: 1.64, 2.35). This was attenuated when adjusting for age (age-adjusted HR = 1.34, 95% CI: 1,12, 1.61) and when adjusting for age, year, deprivation, cancer treatment and comorbidities (adjusted HR = 1.39, 95% CI: 1.14, 1.68). After further adjustment for T stage and N stage, the association was attenuated further (fully adjusted HR = 1.14, 95% CI: 0.92, 1.41). There did not appear to be any association between past use of hormone therapy and acute kidney injury (fully adjusted HR = 1.02, 95% CI: 0.77, 1.34). This pattern was similar for GnRH agonists and GnRH agonists and antagonists combined. However, there was an association between the current use of the GnRH antagonist degarelix and acute kidney injury before and after adjustments (unadjusted HR = 4.60, 95% CI: 2.72, 7.77 and fully adjusted HR = 2.47, 95% CI: 1.38, 4.43).

**Table 2:** Association between hormone therapy and acute kidney injury.

	AKI cases	Person years	Unadjusted HR (95% CI)	*P*	Adjusted^a^ HR (95% CI)	*P*	Fully adjusted^b^ HR (95% CI)	*P*
No hormone therapy use	249	21196	1.00 (ref. cat.)		1.00 (ref. cat.)		1.00 (ref. cat.)	
Current hormone therapy use	253	11595	1.96 (1.64,2.35)	<0.001	1.39 (1.14,1.68)	0.001	1.14 (0.92,1.41)	0.221
Past hormone therapy use	116	9207	1.05 (0.83,1.32)	0.691	1.11 (0.85,1.45)	0.446	1.02 (0.77,1.34)	0.905
GnRH agonist or antagonist current	236	10836	1.98 (1.64,2.37)	<0.001	1.40 (1.15,1.70)	0.001	1.17 (0.94,1.45)	0.151
GnRH agonist or antagonist past	101	8251	1.00 (0.79,1.28)	0.982	1.08 (0.81,1.44)	0.581	1.02 (0.76,1.36)	0.913
GnRH agonists current	221	10588	1.90 (1.58,2.29)	<0.001	1.35 (1.11,1.65)	0.003	1.13 (0.90,1.40)	0.289
GnRH agonists past	97	8204	0.97 (0.76,1.23)	0.777	1.04 (0.78,1.39)	0.797	0.98 (0.73,1.31)	0.87
GnRH antagonist (degarelix) current	15	267	4.60 (2.72,7.77)	<0.001	2.83 (1.62,4.94)	<0.001	2.47 (1.38,4.43)	0.002
GnRH antagonist (degarelix) past	6	189	3.12 (1.38,7.06)	0.006	1.99 (0.84,4.74)	0.119	1.76 (0.73,4.24)	0.208

*AKI* acute kidney injury.

^a^Model contains: age, year, deprivation (in tenths), radiotherapy (as time-varying covariate), radical prostatectomy (as time-varying covariate), medications in the year prior to diagnosis (specifically: aspirin, beta-blockers, ACE inhibitors, ARBs, diuretics, statins, warfarin, digoxin, clopidogrel, dipyridamole, nitrates, insulin, sulfonylureas, metformin and other diabetic medications) and comorbidities prior to diagnosis (specifically: myocardial infarction, congestive heart failure, peripheral vascular disease, stroke, dementia, pulmonary disease, connective tissue disorder, paraplegia, peptic ulcer disease, liver disease, severe liver disease, diabetes and diabetes with complications).

^b^Model contains all terms in 1 as well as T stage and N stage.

### Sensitivity analyses

Table 3 shows sensitivity analyses which in general gave similar results. In particular, associations were similar when restricting the acute kidney injury definition to the main condition within hospital records (current use fully adjusted HR = 1.15, 95% CI: 0.69, 1.90), when altering the duration of the residual effect including all prostate cancer patients (fully adjusted HR = 1.03, 95% CI: 0.88, 1.21). Censoring on radical prostatectomy and radiotherapy or not adjusting for radical prostatectomy and radiotherapy had little impact on the estimates. In patients with localised prostate cancer, a slighted more marked association was seen between current hormone therapy and acute kidney injury (unadjusted HR = 2.23, 95% CI: 1.63, 3.05) which was only partly attenuated when adjusting for stage (adjusted HR = 1.48, 95% CI: 1.06, 2.08), but was largely attenuated after further adjusting for Gleason score (unadjusted HR = 1.23, 95% CI: 0.83, 1.82).

**Table 3:** Sensitivity analyses for the association between hormone therapy and acute kidney injury.

	AKI cases	Person years	Current vs. no hormone therapy use				Past vs. no hormone therapy use			
			Unadjusted HR (95% CI)	*P*	Fully adjusted^a^ HR (95% CI)	*P*	Unadjusted HR (95% CI)	*P*	Fully adjusted^a^ HR (95% CI)	*P*
Main analysis	618	41997	1.96 (1.64, 2.35)	<0.001	1.14 (0.92, 1.41)	0.221	1.05 (0.83, 1.32)	0.691	1.02 (0.77, 1.34)	0.905
Outcome										
Based upon solely primary AKI	119	42882	3.20 (2.10, 4.88)	<0.001	1.15 (0.69, 1.90)	0.594	1.18 (0.68, 2.03)	0.556	0.75 (0.39, 1.42)	0.373
Hospitalisation or death from AKI	628	41997	1.96 (1.64, 2.34)	<0.001	1.14 (0.92,1.40)	0.23	1.02 (0.81, 1.29)	0.852	0.99 (0.75, 1.30)	0.941
Exposure										
Lag of 90 days	578	39356	2.09 (1.73, 2.52)	<0.001	1.23 (0.99, 1.54)	0.062	1.00 (0.78, 1.28)	0.994	0.97 (0.72, 1.29)	0.825
Reducing residual period^b^	618	41997	1.97 (1.64, 2.37)	<0.001	1.14 (0.92, 1.41)	0.231	1.09 (0.87, 1.36)	0.454	1.03 (0.79, 1.35)	0.814
Increasing residual period for GnRH agonist^c^	618	41997	1.81 (1.52, 2.16)	<0.001	1.13 (0.92, 1.39)	0.259	1.05 (0.81, 1.36)	0.718	1.03 (0.76, 1.38)	0.861
Population										
Patients with M stage 0 or X	859	55039	1.91 (1.64, 2.22)	<0.001	1.11 (0.93, 1.32)	0.264	1.09 (0.90, 1.33)	0.387	1.08 (0.86, 1.36)	0.513
Localised prostate cancer	242	24426	2.23 (1.63, 3.05)	<0.001	1.48 (1.06, 2.08)	0.023	1.22 (0.88, 1.71)	0.235	0.98 (0.65, 1.46)	0.913
Localised prostate cancer (additionally adjusting for Gleason score^d^)	242	24426	2.23 (1.63, 3.05)	<0.001	1.23 (0.83, 1.82)	0.294	1.22 (0.88, 1.71)	0.235	0.86 (0.57, 1.30)	0.475
Not restricted to non-metastatic	1352	62868	2.53 (2.24, 2.87)	<0.001	1.03 (0.88, 1.21)	0.696	1.23 (1.02, 1.47)	0.026	1.08 (0.87, 1.32)	0.487
Confounders										
Censoring on prostatectomy or radiotherapy	394	19820	2.35 (1.90, 2.90)	<0.001	1.13 (0.89, 1.44)	0.325	1.32 (0.89, 1.96)	0.171	1.00 (0.67, 1.51)	0.984
Not adjusting for prostatectomy or radiotherapy	618	41997	1.96 (1.64, 2.35)	<0.001	1.06 (0.87, 1.29)	0.58	1.05 (0.83, 1.32)	0.691	0.85 (0.66, 1.08)	0.172
Additionally adjusting for Gleason score^d^	618	41997	1.96 (1.64, 2.35)	<0.001	1.08 (0.83, 1.41)	0.579	1.05 (0.83, 1.32)	0.691	0.91 (0.67, 1.24)	0.566

*ADT* androgen deprivation therapy, *AKI* acute kidney injury.

^a^Model contains the following except where otherwise stated: age, year, deprivation (in tenths), radiotherapy (as time-varying covariate), radical prostatectomy (as time-varying covariate), medications in the year prior to diagnosis (specifically: aspirin, beta-blockers, ACE inhibitors, ARBs, diuretics, statins, warfarin, digoxin, clopidogrel, dipyridamole, nitrates, insulin, sulfonylureas, metformin and other diabetic medications) and comorbidities prior to diagnosis (specifically: myocardial infarction, congestive heart failure, peripheral vascular disease, stroke, dementia, pulmonary disease, connective tissue disorder, paraplegia, peptic ulcer disease, liver disease, severe liver disease, diabetes and diabetes with complications), T stage and N stage.

^b^Assuming a residual effect duration of 45 days for GnRH and degarelix and 15 days for anti-androgens and estrogens.

^c^Assuming a residual effect duration of 365 days for GnRH, 90 days for degarelix and 30 days for anti-androgens and estrogens.

^d^Model contains all variables in ^a^ plus Gleason score.

Finally, a similar association was observed when any use of hormone therapy was investigated (unadjusted HR = 1.56, 95% CI: 1.32, 1.84 and fully adjusted HR = 1.11, 95% CI: 0.91, 1.36) or any use of GnRH agonists was investigated (unadjusted HR = 1.49, 95% CI: 1.26, 1.77 and fully adjusted HR = 1.09, 95% CI: 0.88, 1.34). Patients using over 365 DDDs of androgen deprivation therapy, compared with non-users, had a more marked increase in acute kidney injury than patients using less than 365 DDDs, compared with non-users, before adjustments (unadjusted HR = 1.21, 95% CI: 0.98, 1.49 and unadjusted HR = 1.96, 95% CI: 1.62, 2.38, respectively). However, these associations were attenuated after adjustments (fully adjusted HR = 1.02, 95% CI: 0.81, 1.28 and fully adjusted HR = 1.23, 95% CI: 0.97, 1.56, respectively).

## Discussion

In our study, patients with non-metastatic prostate cancer were at higher risk of hospitalisation with acute kidney injury compared with population-based controls. However, in prostate cancer patients, hormone therapy use and specifically GnRH agonist use was not associated with a higher risk of hospitalisation with acute kidney injury, after adjusting for confounders.

The only previous study to investigate acute kidney injury and current hormone therapy use [5] observed almost a 150% increase (OR = 2.48, 95% CI: 1.61, 3.82) which is not consistent with our estimate of a 14% increase. The reason for the difference in findings is unclear but could partly reflect the time periods (they included cases diagnosed 1997–2009), differences in adjustments (they adjusted for smoking but not stage) or differences in the identification of prostate cancer patients.

A previous US study [6] investigating any use of GnRH agonists observed a comparatively small 24% increase in acute kidney injury risk (HR = 1.24, 95% CI: 1.18, 1.31). Notably, they did not investigate current use or a dose response, as data on the timing of androgen deprivation therapy were not available, and their study only included men over 66 years of age. In our analysis of ever use of GnRH agonists, we observed a non-significant 9% increase (HR = 1.09, 95% CI: 0.88, 1.34), but we cannot rule out small increases in risk based upon our CI.

Overall, our findings do not appear to support the hypothesis that hormone therapy markedly increases the risk of acute kidney injury [5, 6] and should provide some reassurance to clinicians prescribing, and patients, taking androgen deprivation therapy. The observed increased risk of acute kidney injury with the GnRH antagonist degarelix is difficult to interpret as it was one of a number of analyses, based upon relatively few acute kidney injury events and because degarelix users are likely to have more advanced prostate cancer. Notwithstanding these caveats, this finding merits further research, particularly as this is the first investigation of this association.

Our study has various strengths. We assembled a large cohort of prostate cancer patients with detailed information on hormone therapy use and confounders including cancer stage. Acute kidney injury was ascertained from Scottish hospital records (SMR01), and although these records have been shown to have high accuracy for a range of conditions [12], misclassification is possible. The rate of acute kidney injury in our prostate cancer cohort (15 per 1000 person years) was higher than the previous UK study [5] (6 per 1000 person years) but lower than a US study [6] (27 per 1000 person years). The definition we used for acute kidney injury (of N17 as a main or secondary condition within hospital admissions data) has been previously validated with 95% of patients identified by this definition meeting Kidney Disease: Improving Global Outcomes criteria for acute kidney injury [14]. Furthermore, a separate analysis was conducted with acute kidney injury based solely on the main condition within hospital admissions data and similar findings were observed.

There is the possibility of residual confounding by incompletely recorded variables (e.g. Gleason score and T stage were 6% and 3% missing, respectively; and, the completeness of capture of certain treatments, for instance radiotherapy, is unclear) or unavailable variables (such as prostate-specific antigen, smoking, alcohol intake, ureteroscopy and nephrostomography). Also, data on prostate cancer progression during follow-up were not available, but this seems unlikely to explain our null results as patients who progress are more likely to receive hormone therapy and therefore may be at higher risk of acute kidney injury due to local invasion [6]. Hormone therapy use was determined from dispensing records but medication adherence cannot be confirmed. However, the limited evidence that exists suggest high adherence to oral androgen deprivation therapies [19]. Finally, patients receiving hormone therapies may have increased exposure to health care professionals increasing the likelihood of identifying acute kidney injury.

## Data Availability

Data were obtained and analysed, within a virtual safe haven, under strict licence conditions from the National Health Service National Services Scotland which do not permit data sharing. However, a researcher would be able to reconstruct these datasets and replicate these analyses after obtaining similar approvals from the National Health Services National Services Scotland.
